# The benefits of Qigong exercise for symptoms of fatigue: A protocol for systematic review and meta-analysis

**DOI:** 10.1097/MD.0000000000023983

**Published:** 2021-01-22

**Authors:** Rui Wang, Xueyan Huang, Yeqi Wu, Dai Sun

**Affiliations:** a Hangzhou Hospital of Traditional Chinese Medicine; b Zhejiang University of Traditional Chinese Medicine, Hangzhou, China.

**Keywords:** fatigue, meta-analysis, protocol, qigong exercise, systematic review

## Abstract

**Background:**

About 5% to 20% of the general population suffers from continuous fatigue, which is also one of the most frequent symptoms seen in primary medical care. Fatigue, if not effectively relieved, may have an adverse effect on the quality of life and treatment of patients. Qigong is a safe and comfortable exercise popular in China and among the Ethnic Chinese, and is a complementary therapy used to treat fatigue, such as cancer related fatigue and chronic fatigue syndrome. It appears promising but lacks convincing evidence; thus, necessitating further investigation.

**Methods:**

Eight databases (PubMed, Web of Science, Embase, Cochrane Library, China Biology Medicine disc, China National Knowledge Infrastructure, WanFang and VIP Data Knowledge Service Platform) will be searched from inception to November 30, 2020 for randomized controlled trials published in English or Chinese investigating the outcomes of the Qigong exercise (QE) for symptoms of fatigue and quality of life. We will also include additional references of relevant published studies by manually retrieving reference lists of included literature and previous relevant reviews. For articles that meet the inclusion criteria, 2 reviewers will independently conduct the study selection, data extraction, and risk of bias assessment. The results of the outcome measures will be reported in risk ratios for dichotomous data and standard mean differences for continuous data. If necessary, we will also perform subgroup analyses and analyze the causes.

**Results:**

The results of this review will be submitted to a journal for publication.

**Conclusion:**

This systematic review protocol aims to analyze whether QE is effective in treating fatigue associated with various disease.

**Registration:**

INPLASY2020110133.

## 1 Introduction

### 1.1 Rationale

Fatigue, a subjective feeling and symptom, is the embodiment of a complex physiological, psychological, and pathological phenomenon in which the body's physical strength or energy is weakened or lost.^[1]^ For healthy people, fatigue is a positive signal of physical, or mental stress, which can be alleviated by some reasonable rest. In contrast, subjective sensations of fatigue, characterized by a persistent sense of it, are attributed to illness or negative symptoms and can not be easily improved by some comfortable sleep.

Well-known etiological factors of cumulative fatigue that have been reported include cancer,^[2]^ cancer-related chemotherapy^[3]^ or surgery,^[4]^ multiple sclerosis,^[5]^ and depression^[6]^ among others. Fatigue can also be seen in independent chronic fatigue syndrome,^[7]^ and may be a precipitating factor of death due to cardiac arrest,^[8]^ myocarditis,^[9]^ and other diseases.^[10,11]^ Hence, the underlying mechanism of fatigue may be multi-factorial and multidimensional, and at the same time, the absence of a consensus on the definition makes it a continuously underestimated symptom. Apart from the basic disease treatment, symptomatic treatment, and nonpharmacological interventions, fatigue does not have any specific drug for its management.^[12,13]^ Additionally, some studies have regarded fatigue as an independent disease predictor^[14]^; therefore, management and prevention of fatigue has become essential in recent times, to maintain a better quality of life.

As the patients’ expectation of a better quality of life keeps increasing, we often choose complementary and alternative medicine as the approach for fatigue-related symptom management.^[15,16]^ Qigong exercise (QE) is a simple, secure, and cost-effective treatment, which has amassed a large following in China.^[17,18]^ Qigong, a kind of mind-body exercise with traditional Chinese characteristics, addresses physiological reconditioning with a lighter amount of exercise, so that you can have a balance between activity and rest. At the same time, its main goal is not only to improve physiological function, but also to calm the patients emotionally and quicken their psychological recovery.^[19,20]^

### 1.2 Objectives

According to our preliminary search, we found that there is no independent systematic review and meta-analysis of QE alone or in combination with other treatments to improve the symptoms of fatigue. Hence, in this study, we are aiming to assess whether QE is safe and effective in the management of fatigue caused by different primary diseases along with an assessment of which type of QE has a higher frequency of utilization.

## 2 Methods

### 2.1 Study registration

This protocol has been registered on INPLASY (https://inplasy.com) and the registration number is INPLASY2020110133. The study follows the preferred reporting items for systematic reviews and meta-analyses protocol (PRISMA-P) statement guidelines.^[21]^

### 2.2 Eligibility criteria for study selection

#### 2.2.1 Types of study

Randomized controlled trials (RCTs) with no limitations on blinding published in English or Chinese up to November 30, 2020 will be included. If more than 1 RCT have been carried out on the same population, the most integrated one will be included in the review.

#### 2.2.2 Types of participants

Patients with primary disease-related symptoms of fatigue will be included, regardless of the underlying disease, age, gender, education, ethnicity, and occupation. However, if the patients included in the literature experience serious complications, cognitive communication disorders, or are pregnant, the study will be excluded.

#### 2.2.3 Types of interventions

1. *Observation interventions:* Qigong-related exercises alone (any form of Qigong category, including Baduanjin, Yijinjing, and Wuqinxi) or Qigong combined with other treatments will be the main intervention in the observation group. There is no limit to the duration and frequency of therapy.
2. *Control interventions:* There is no exclusion based on the comparator method for this review, and the patients may be treated with any type of control group including exercise, stretching, sham Qigong, waiting list control, or other treatments.

#### 2.2.4 Outcomes and prioritization

1. *Primary outcomes:* Medical scales, which are mainly used to evaluate the severity, duration, frequency, improvement, and symptoms of fatigue related to the primary disease, will be accepted as the main types of outcome measures. (For example, Flinders Fatigue Scale, Functional Assessment of Chronic Illness Therapy-Fatigue, Visual Analogue Scale-Fatigue, Multidimensional Fatigue Inventory).
2. *Additional outcomes:* The quality of life assessment scale for social well-being, physical functioning, and mental functioning.

### 2.3 Information sources

The following electronic databases will be searched from their inception to November 30, 2020: PubMed, Web of Science, Embase, Cochrane Library, China Biology Medicine disc, China National Knowledge Infrastructure, WanFang, and VIP Data Knowledge Service Platform. Furthermore, reference lists of included literature and previous relevant reviews will be manually retrieved to further locate additional trials and identify additional articles that meet our inclusion criteria. The search is limited to English or Chinese-language papers, with no other restrictions applied. Detailed search strategies for each electronic database have been developed by the review team members. The English-language search strategy contains relevant terms will be based on the concepts:

1. “Fatigue” or “Malaise” or “Tiredness” AND
2. “Qigong” AND
3. “Randomized Clinical Trial”.

In the Chinese databases, we will use equivalent search terms. The search strategy for PubMed and China National Knowledge Infrastructure is attached as a word document. Search tactics for PubMed are shown in Table 1.

**Table 1:** Search strategy for PubMed.

Search	Query
#1	fatigue[mesh] OR “muscle fatigue”[mesh] OR “fatigue syndrome, chronic”[mesh] OR fatigue^∗^[tiab] OR malaise[tiab] OR tired^∗^[tiab] OR exhaust^∗^[tiab] OR fatigability[tiab] OR weary[tiab] OR weariness[tiab] OR listlessness[tiab] OR lassitude[tiab] OR asthenia[tiab] OR asthenic[tiab] OR lacklustre[tiab]
#2	Qigong[mesh] OR Qigong[tiab] OR Qi-gong[tiab] OR “Qi gong”[tiab] OR “Chi chung”[tiab] OR “Chi gong”[tiab] OR “Chi Kung”[tiab] OR “Qi Kung”[tiab] OR “Jhi gong”[tiab] OR “Chi gung”[tiab] OR “Qi chung”[tiab] OR “Ch’i kung”[tiab] OR “Kung ch’i”[tiab] OR Baduanjin[tiab] OR Yijinjing[tiab] OR “Yi jinjing”[tiab] OR Wuqinxi[tiab] OR “Wu qin xi”[tiab]
#3	(“randomized controlled trial”[pt] OR “controlled clinical trial”[pt] OR randomized[tiab] OR placebo[tiab] OR “clinical trials as topic”[mesh:noexp] OR randomly[tiab] OR trial[ti]) NOT (animals[mh] NOT (humans[mh] AND animals[mh]))
#10	#1 AND #2 AND #3

### 2.4 Study records

*Data management:* The search results which are extracting from the abovementioned electronic databases and additional trials will be managed through Endnote X9. *Selection process:* Two reviewers (WR and W-YQ) will independently exclude articles based on the eligibility criteria mentioned above by the following steps:

1. Exclude duplicate literature,
2. reviewing the titles and abstracts,
3. acquiring the full text for screening.

**Figure 1:**
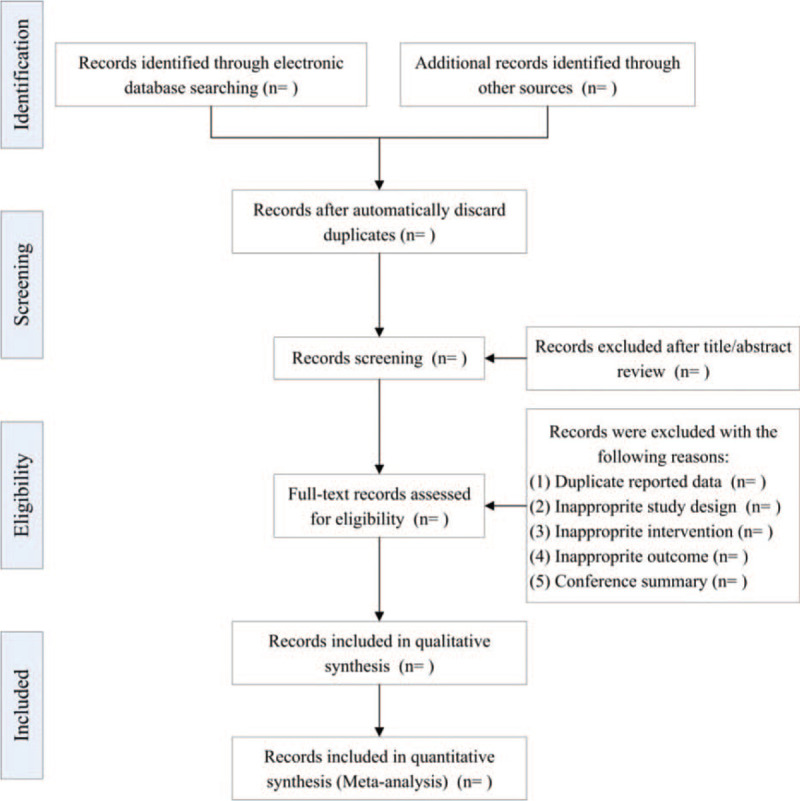
Flow diagram for study selection.

Discrepancy in the eligibility of an article will be solved by discussion and arbitrated by a third reviewer (SD). The process of the study selection is summarized in a PRISMA-P flow diagram (Fig. 1).

### 2.5 Data collection process and data items

Two investigators (WR and H-XY) will independently extract information from the selection of articles, which will be collected and sorted by the unified data statistics table; including study characteristics (reference ID, first author, publication year, and sample size); baseline data (age, gender, and ethnicity); primary disease conditions; methodology of the trial (including but not limited to randomization, allocation concealment, and blinding and its methods); characteristics of interventions in the experimental and control group (the detailed description of manual therapies, including dosage, frequency, and course of treatment); measurement of fatigue and quality of life (quantitative data reported, mean and standard deviations, and confidence intervals); adverse events; follow-up period; and funding. We will depend on the preliminary discussion to resolve disagreement on data extraction. In the event of the persistence of a disagreement, the third investigator (SD) will mediate.

### 2.6 Risk of bias assessment in individual studies

Two researchers (WR and H-XY) will independently evaluate the quality of the selected studies according to the Cochrane Collaboration's tool for randomized controlled trials.^[22]^ Items will be assessed in 3 categories: Low risk of bias, unclear bias, and high risk of bias. The following characteristics will be evaluated: Random sequence generation (selection bias), Allocation concealment (selection bias), Blinding of participants and personnel (performance bias), Incomplete outcome data (attrition bias), Selective reporting (reporting bias), and other biases resulting from these questions will be graphed and assessed using the Review Manager 5.4. Any disagreements will be resolved by discussion or with the help of the third author (SD).

### 2.7 Data synthesis

Efficacy data which conform to the same outcomes in the populations will be synthesized and statistically analyzed in STATA software for Windows 10. Risk ratio (RR) with 95% confidence intervals (CIs) will be utilized to dichotomous data. For continuous data measured on the same measurement, the mean difference and 95% CI will be used for effect evaluation, while the standard mean difference will be used for results measured on different measurements. Random effects models (weighted by the inverse of the variance) will be used. A continuity correction will also be used for cells with zero values. *P* value <.05 will be considered statistically significant.

### 2.8 Assessment of heterogeneity

Between-study heterogeneity will be assessed using the χ^2^ tests and *I*^2^ test. According to the Cochrane handbook, the *I*^2^ will be considered substantial (>60%), moderate (30%–60%), and nonimportant (<30%). If the *I*^2^ statistic was 50% or higher, indicating the presence of heterogeneity. We will screen the reasons for the extreme heterogeneity.

### 2.9 Subgroup analysis

If we acquire sufficient data, we plan to perform the analysis on the following subgroups of interest: primary diseases, QE type, and quality of studies. We will assess subgroup effects with an *I*^2^ statistic for interpreting the heterogeneity.

### 2.10 Sensitivity analysis

If there is sufficient data for analysis, the sensitivity analysis will be performed to identify the results of the meta-analysis by excluding each of the individual studies.

### 2.11 Assessment of meta-bias (es)

When more than 10 studies are included, publication bias will be judged by a funnel plot for meta-analysis and quantified by the Egger method.

### 2.12 Confidence in cumulative evidence

The Grading of Recommendations Assessment, Development and Evaluation (GRADE) system will be used to evaluate the quality of the evidence for each outcome, which is divided into the following categories: high, moderate, low, or very low-quality evidence.^[23]^

### 2.13 Ethics and dissemination

The development of the research question and outcome measures according to patients’ priorities, experience, and preferences were reported in the included published clinical trials. Patients and public will not participate in this study.

## 3 Discussion

With the rapid development of an industrialized society, fatigue has become a common phenomenon, and is expected to be an objective indicator of quality of life to assess people's physical and mental functioning, disease prognosis, and pain. In fact, there appears to be a consistent overlap between fatigue, pain, depression, and anxiety.^[24,25]^ Our focus is not on making the presence of fatigue sufficiently distinctive from other symptoms, rather, preventing it from a vicious circle of mutual influence.

Therefore, new requirements have been put forward for the treatment of the symptoms of fatigue, which include making the treatment easy, safe, effective, and comfortable; meanwhile, the search for a universal therapy to improve fatigue continues. QE may be a better strategy to address this problem, especially in China and among the ethnic Chinese. We will conduct this review when a sufficient number of appropriate trials are available, and all review procedures will be performed in accordance with PRISMA-P to ensure that the clinical information provided to physicians is of high quality.

However, there are certain limitations to this review which mainly includes different forms of Qigong, sects, intervention frequency, and course of treatment. The evaluation of fatigue needs to rely on subjective indicators, and their measures may lead to inconsistent results when assessed by different personnel. In addition, this review will only include studies published in English or Chinese due to the language restriction, which might cause a potential risk of publication bias. Therefore, we will use subgroup analysis to reduce inconsistencies. However, we believe that the purpose of all QE is the same, which is to regulate the body and mind of patients through traditional methods, so a certain degree of bias is acceptable.

## Author contributions

**Conceptualization:** Dai Sun.

**Data curation:** Rui Wang.

**Funding acquisition:** Rui Wang.

**Methodology:** Rui Wang, Xueyan Huang.

**Software:** Xueyan Huang, Yeqi Wu.

**Supervision:** Dai Sun.

**Writing – original draft:** Rui Wang.

**Writing – review & editing:** Dai Sun.
